# Early-Life Maternal Deprivation Predicts Stronger Sickness Behaviour and Reduced Immune Responses to Acute Endotoxaemia in a Pig Model

**DOI:** 10.3390/ijms21155212

**Published:** 2020-07-23

**Authors:** Roberto Brückmann, Margret Tuchscherer, Armin Tuchscherer, Ulrike Gimsa, Ellen Kanitz

**Affiliations:** 1 Institute of Behavioural Physiology, Leibniz Institute for Farm Animal Biology (FBN), 18196 Dummerstorf, Germany; brueckmann@fbn-dummerstorf.de (R.B.); mtuchsch@fbn-dummerstorf.de (M.T.); 2 Institute of Genetics and Biometry, Leibniz Institute for Farm Animal Biology (FBN), 18196 Dummerstorf, Germany; atuchsch@fbn-dummerstorf.de

**Keywords:** maternal deprivation, social isolation, early-life adversity, innate immunity, sickness behaviour, HPA axis, *Sus scrofa*, sex differences

## Abstract

Early-life adversity may have programming effects on neuroendocrine and immune adaptation mechanisms in humans and socially living animals. Using a pig model, we investigated the effect of daily 2-h maternal and littermate deprivation from postnatal days 2–15, either alone (DA) or in a group of littermates (DG) on the neuroendocrine, immunological and behavioural responses of piglets challenged with the bacterial endotoxin lipopolysaccharide (LPS) on day 42. LPS increased plasma concentrations of cortisol, tumour necrosis factor-alpha (TNF-α), interleukin-6 (IL-6) and interleukin-10 (IL-10) and induced typical signs of sickness in all piglets. DA+DG piglets showed stronger signs of sickness compared to control (C) piglets. Plasma TNF-α concentrations were significantly lower in DA+DG males. In addition, the TNF-α/IL-10 ratio was significantly lower in DA than in DG and C males. Gene expression analyses showed lower hypothalamic TNF-α mRNA expression and diminished mRNA expression of the mineralocorticoid receptor (MR) and IL-10 in the amygdala of DA+DG piglets in response to LPS. Interestingly, males showed a higher MR- and a lower IL-10 mRNA expression in the amygdala than females. The present data suggest that repeated maternal deprivation during early life may alter neuroendocrine and immune responses to acute endotoxaemia in a sex-specific manner.

## 1. Introduction

Early-life adversity increases the risk of a multitude of psychological, neurological and physiological problems in adulthood, including mood disorders, cognitive deficits, cardiovascular disease, cerebrovascular infarction and cancer [1,2]. Adversity at an early age has the greatest impact on the individual’s development in the perinatal, adolescent and puberty phases, when the developing brain is particularly vulnerable to the programming effects of stress [3]. During these critical phases, acute or chronic stress can trigger long-lasting or even permanent changes in the metabolism, central nervous system and immune system of young individuals [4,5]. Pigs (*Sus scrofa domesticus*) are an ideal model species for studying the programming effects of early-life adversity [6,7] because they share similarities in brain anatomy and neurodevelopment with humans [8,9] and their immune system resembles that of humans to a large extent in anatomy, function and gene expression [10,11,12]. In addition, they raise fewer ethical questions than primate models. Like humans but unlike rodents, pigs show a robust response of the hypothalamic-pituitary-adrenal (HPA) axis to stressors throughout their early postnatal period [13].

There is growing evidence that exposure to psychosocial stressors in pig husbandry may have negative impacts on health and welfare [7,14]. Psychosocial stress activates the HPA axis resulting in the release of glucocorticoids, which influence a wide range of biological functions including cytokine secretion of immune cells and behaviour and are generally considered to be immunosuppressive [15,16]. It has been shown that acute stress exposure potentiates the peripheral and central production of pro-inflammatory cytokines in response to lipopolysaccharide (LPS), while chronic stress exposure diminishes their release [17]. Cytokines such as TNF-α, IL-6 and IL-1 are capable of stimulating several CNS functions including sleep, fever or even the release of stress hormones [18]. Although the mechanisms of the neuroendocrine and immunological interactions are not yet fully elucidated, there is evidence that psychosocial stress causes sensitisation of inflammatory processes [19,20,21].

In animal husbandry, pigs are subject to the loss of social relations during weaning and regrouping. It is known that such a disruption of social bonds in socially living animals is a strong psychosocial stressor [14]. Moreover, psychosocial stress has been shown to alter cytokine concentrations with consequences on sickness behaviour [22,23,24]. Maternal and littermate deprivation is commonly used to study psychosocial stress [25,26,27]. This type of psychosocial stress can cause neurobiological changes, which may be sex dependent [28,29,30]. In pigs, maternal and littermate deprivation may cause dysregulation of the neuroendocrine and immunological balance and may increase susceptibility to disease [21]. 

The administration of LPS is a common model to study the effects of a bacterial infection in animals and humans. LPS is part of the cell wall of Gram-negative bacteria and is responsible for the stimulation of the innate immune system by binding to the receptors CD14 and TLR4 [31,32]. By stimulating pro-inflammatory cytokine production, LPS is a potent inducer of non-specific symptoms of sickness such as fever, loss of appetite or lower general activity [22]. Interestingly, LPS-induced cytokine responses after psychosocial stress were found to be gender dependent in humans [33].

There are many studies dealing with the effects of stress on the immune system or neuroendocrine regulation. However, most of these studies have been conducted in rodent models and little is known about the complex programming effects of early postnatal psychosocial stress on the susceptibility of pigs to diseases. Previous studies by our group found that repeated 2h-daily social isolation of piglets from days 3 to 11 of life had long-term effects on HPA-axis activity and immune-brain circuitry [34] as well as on neuroendocrine and immune responses to LPS [35]. Further studies showed that even a single 4-h social isolation during early childhood altered neuroendocrine stress hormones, stress-related gene expression and immune functions in piglets [36,37]. Social support reduced effects of this social isolation [38,39]. Based on these findings, we established a new pig model with the aim to study programming effects of repeated maternal and littermate deprivation on behavioural, neuroendocrine and immune responses to LPS with or without social support by a group of littermates. We hypothesise that repeated psychosocial stress during early life has a profound impact on the neuroendocrine and immunological responses to later-life challenges and assume that stressors experienced in a group would be perceived as less stressful than the same stressors experienced alone. To test these hypotheses, piglets were separated from their mothers and littermates either alone or together with a group of littermates for 2 h daily over a period of two weeks. Four weeks later, the piglets were challenged by LPS to assess the effects of different psychosocial treatments on sickness behaviour and neuroendocrine-immune interactions.

## 2. Results

### 2.1. Sickness Behaviour

Piglets received LPS injections on the 42nd day of life and were killed 24 h later. Prior to this, from the 2nd to the 15th day of life, the piglets either experienced maternal and littermate deprivation alone (DA) or in a group of five littermates (DG) for 2 h daily, or they were kept as controls with their mothers in their litters (C). 

Table 1 shows all the results of the statistical analysis of the frequencies of clinical signs of disease in response to LPS application. ANOVA indicated a significant main effect of the deprivation treatment on the sickness parameters panting (F_2,22_ = 3.73, *p* < 0.05) and somnolence (F_2,82_ = 3.61, *p* < 0.05). Additionally, statistical analysis revealed an effect of the interaction treatment × time × sex interaction on vomiting (F_10,273_ = 1.94, *p* < 0.05). 

**Figure 1:**
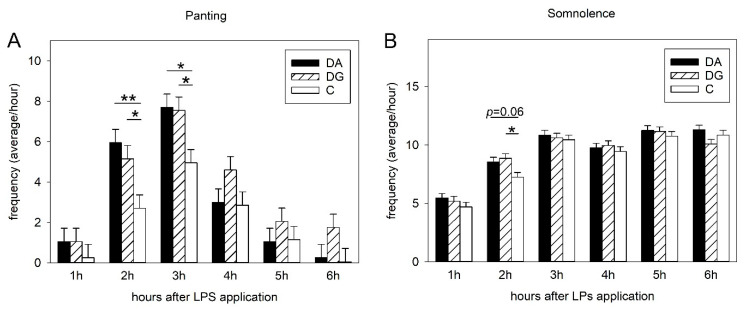
Sickness symptoms of panting (**A**) and somnolence (**B**) after LPS challenge in the three treatment groups of DA (deprivation alone), DG (deprivation with a group of littermates) and C (control, no deprivation). Significant differences are indicated by asterisks (* *p* < 0.05; ** *p* < 0.01; *n* = 20 pigs per treatment group).

The Tukey-Kramer test indicated significantly higher frequencies of panting in the DA (deprivation alone) and DG (deprivation in a group of littermates) treatment groups compared to the controls (C), 2 h (DA vs. C, *p* < 0.01; DG vs. C, *p* < 0.05) and 3 h (*p* < 0.05) after LPS application (Figure 1A). Additionally, the frequencies of somnolence were significantly higher in DG than in C pigs (*p* < 0.05) 2 h after LPS application, and tended to be higher for DA compared to C pigs (*p* = 0.06; Figure 1B). 

**Figure 2:**
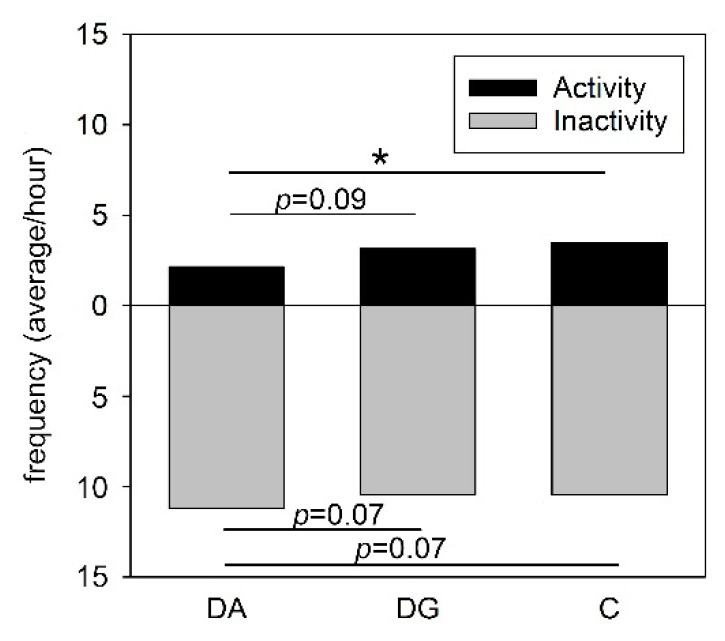
Activity and inactivity of the three treatment groups of DA (deprivation alone), DG (deprivation with a group of littermates) and C (control, no deprivation) 1 h after LPS application. Significant differences are indicated by asterisks (* *p* < 0.05; *n* = 20 pigs per treatment group).

One hour after LPS application, the general activity of DA pigs was significantly lower than that of C pigs (*p* < 0.05) but the difference between DA and DG pigs failed to reach statistical significance (*p* = 0.09). In turn, the inactivity of the DA pigs tended to be higher compared to DG and C pigs (both, *p* = 0.07; Figure 2). 

### 2.2. Cytokine and Hormone Analysis

Statistical analysis (ANOVA) revealed a significant main effect of time on all measured parameters (all *p* < 0.001) but not for treatment or sex as shown in Table 2. However, the plasma TNF-α concentrations of the piglets in response to the LPS challenge were affected by the treatment × time (F_8,186_ = 2.2, *p* < 0.05) and treatment × sex × time (F_8,186_ = 2.7, *p* < 0.01) interactions.

**Figure 3:**
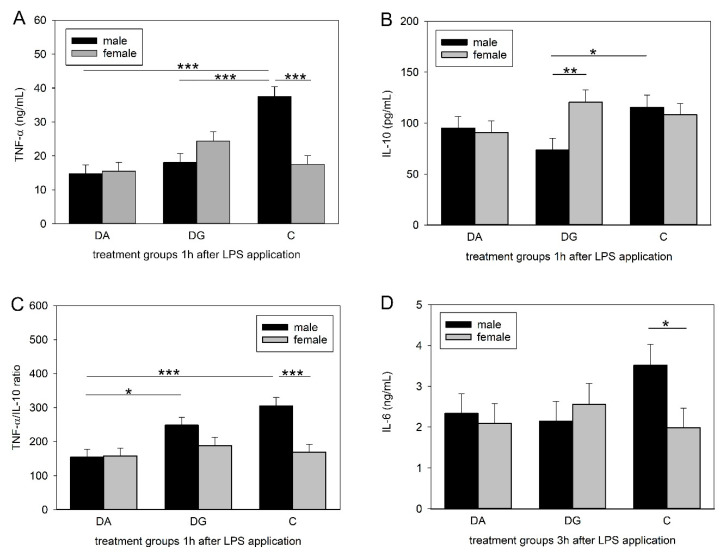
Plasma concentrations of TNF-α (**A**) and IL-10 (**B**) and the TNF-α/IL-10 ratio (**C**) 1 h after LPS application and plasma concentration of IL-6 (**D**) 3 h after LPS application in male and female piglets of the three treatment groups of DA (deprivation alone), DG (deprivation with a group of littermates) and C (control, no deprivation). Significant differences are indicated by asterisks (* *p* < 0.05; ** *p* < 0.01; *** *p* < 0.001; *n* = 8 pigs per treatment group and sex).

As shown in Table 2, TNF-α and IL-10 reached maximum values 1 h after LPS application while IL-6 and cortisol reached their maximum 3 h after LPS administration. At this time, the Tukey-Kramer test indicated significant differences between the three treatment groups (Figure 3). The LPS-induced increase in the plasma TNF-α concentration was significantly diminished in the male piglets of both stress groups compared to the male controls (DA, DG vs. C, *p* < 0.001; Figure 3A). Moreover, the plasma TNF-α concentrations of control piglets were significantly higher in males than in females (*p* < 0.001). The plasma concentrations of IL-10 were significantly lower (*p* < 0.05) in male DG piglets compared to male C piglets and significantly lower compared to the females of the DG piglets (*p* < 0.01; Figure 3B). The Tukey-Kramer test revealed a significantly lower TNF-α/IL-10 ratio in male DA piglets compared to male DG and C piglets (DA vs. DG, *p* < 0.05; DA vs. C, *p* < 0.001), as shown in Figure 3C. Significant differences were also found for the IL-6 plasma concentration, with male control piglets having a higher concentration than the female control piglets (*p* < 0.05; Figure 3D). 

### 2.3. Brain mRNA Expression 

#### 2.3.1. Hypothalamus

ANOVA showed that TNF-α mRNA expression in the hypothalamus of piglets challenged with LPS was significantly affected by social treatment (F_2,22_ = 7.50, *p* < 0.01) and, in tendency, by sex (F_1,22_ = 3.58, *p* = 0.07). Social treatment had no significant effect on MR, GR, CRHR1, CRHR2, IL-6 and IL-10 mRNA expression levels (Table 3) but tended to have a sex effect on MR mRNA expression (F_1,22_ = 3.98, *p* = 0.06).

**Figure 4:**
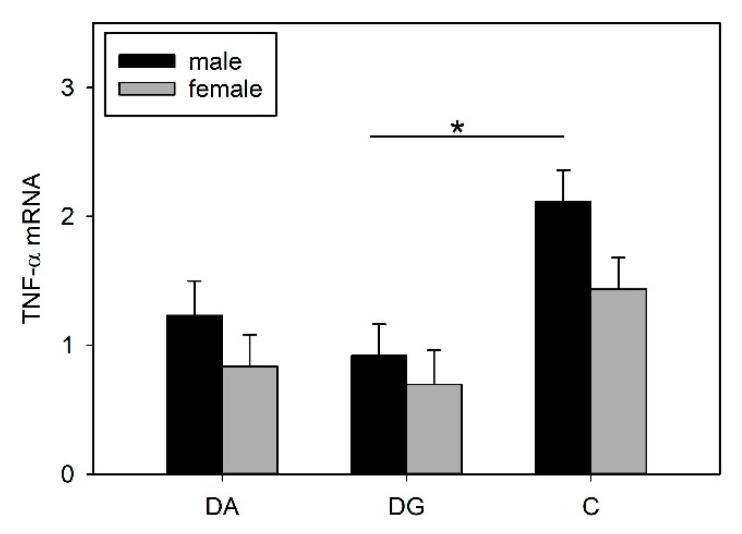
Hypothalamic TNF-α mRNA expression of DA (deprivation alone), DG (deprivation with a group of littermates) and C (control, no deprivation) piglets 24 h after LPS application. Data are expressed as arbitrary units after normalisation to ACTB and TBP mRNA expression as endogenous reference genes and represent the LS means ± SE. Significant differences are indicated by asterisks (* *p* < 0.05; *n* = 7 pigs per treatment group and sex).

The Tukey-Kramer test revealed significantly lower TNF-α mRNA expression in socially deprived piglets than in the control pigs (DA vs. C, *p* < 0.05; DG vs. C, *p* < 0.01; Table 3). With regard to sex, the male piglets of the C group had a significantly higher expression of TNF-α compared to the DG group (*p* < 0.05), whereas this effect was not present for the female pigs (Figure 4).

#### 2.3.2. Amygdala

Statistical analysis revealed significant effects of the deprivation treatment on the mRNA expression of MR (F_2,23_ = 9.74, *p* < 0.001), GR (F_2,23_ = 3.72, *p* < 0.05) and IL-10 (F_2,22_ = 7.06, *p* < 0.01) in response to LPS. Furthermore, there was a main effect of sex on the mRNA expression of MR (F_1,23_ = 7.0, *p* < 0.05), the MR/GR ratio (F_1,23_ = 7.23, *p* < 0.05) and IL-10 (F_1,22_ = 4.67, *p* < 0.05) as well as a significant effect of the treatment × sex interaction for IL-10 (F_2,22_ = 4.28, *p* < 0.05; Table 4). There were no other significant effects on the mRNA expression of the investigated genes (Table 4).

**Figure 5:**
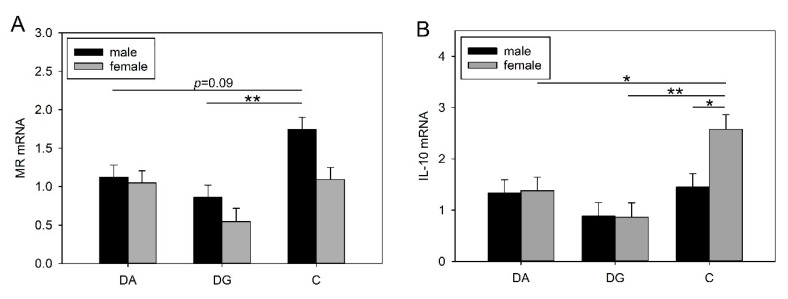
MR (**A**) and IL-10 (**B**) mRNA expression in the amygdala of DA (deprivation alone), DG (deprivation with a group of littermates) and C (control, no deprivation) piglets, 24 h after LPS application. Data are expressed as arbitrary units after normalisation to ACTB and TBP mRNA expression as endogenous reference genes and represent the LS means ± SE. Significant differences are indicated by asterisks (* *p* ≤ 0.05; ** *p* < 0.01; *n* = 7 pigs per treatment group and sex).

Both treatment groups had lower MR mRNA expression compared to the control, but this difference was only statistically significant for the DG piglets (Table 4). Figure 5A shows that this effect was mainly caused by the males. Here, male DG piglets showed significantly lower MR mRNA expression (*p* < 0.01) and male DA piglets showed a trend towards lower MR mRNA expression (*p* = 0.09) compared to male C piglets. In addition, MR mRNA expression was significantly higher in male piglets than in females (males: 1.24 ± 0.09; females: 0.90 ± 0.09; *p* < 0.05), and the MR/GR ratio was also higher in males than in females (males: 1.18 ± 0.12; females: 0.72 ± 0.13; *p* < 0.05). 

Furthermore, the deprivation treatment also caused a decrease in IL-10 mRNA expression in both groups, with a significant effect for the DG compared to C piglets (*p* < 0.01; Table 4). In contrast to all previous results, this alteration was only found in females and not in males. As shown in Figure 5B, IL-10 mRNA expression was significantly lower in female DA and DG piglets than in controls (DA vs. C, *p* ≤ 0.05; DG vs. C, *p* < 0.01), and female control piglets displayed significantly higher mRNA expression of IL-10 than male controls (*p* < 0.05). Overall, females showed significantly higher IL-10 mRNA expression than males (females: 1.61 ± 0.18; males: 1.22 ± 0.17; *p* < 0.05).

## 3. Discussion

This study addresses the complex physiological impact of early-life adversity, modelled by maternal and social deprivation treatments in piglets on their behavioural and physiological responses to an acute endotoxin challenge. Our results demonstrate that repeated stress exposure during the postnatal period causes significant changes in the immunological and neuroendocrine responses to LPS application later in life. Early-life stress enhanced signs of sickness, diminished the cytokine release and modified the expression of HPA axis-regulating genes. However, the neuroendocrine and immune responses were sex dependent. 

Stimulation of the immune system by the endotoxin LPS is often used as a model for bacterial infection. In this study, piglets were injected with LPS, which caused serious sickness symptoms in all pigs such as somnolence, shivering and vomiting, and induced a profound increase in peripheral cytokine and cortisol concentrations. This response was time dependent as the onset of the sickness symptoms reached its peak after approximately 2 to 3 h and slowly declined afterwards. These findings are consistent with results from previous studies, where LPS was shown to activate the HPA axis, to induce cytokine secretion and to evoke a multitude of signs of sickness [35,40,41,42,43]. However, in the present study, social deprivation aggravated the signs of sickness in the pigs of both deprivation groups in response to LPS application compared to controls. In line with this, deprived pigs showed a lower activity compared to the controls. Reduced locomotor activity is a well-known symptom of infections and is important for the animals in order to save energy for the immune system to fight the infection [44]. Thus, a lower activity may indicate that the maternally deprived pigs were more seriously affected by the endotoxin than the control pigs. 

LPS stimulates the innate immune system by binding to TLR4 receptors, which are present on the surface of macrophages [31]. This stimulation activates the transcription factor NF-кB, a protein complex that is crucial for the production of pro-inflammatory cytokines [45] such as IL-1, IL-6 and TNF-α, which are important for the response to bacterial infections and the induction of sickness behaviour [46]. In pigs, stimulation of the immune system with LPS causes a strong elevation of peripheral TNF-α and IL-6 concentrations [47,48], which is consistent with our findings. However, prior deprivation treatment caused a significantly diminished increase in peripheral TNF-α concentrations in DA and DG pigs. Interestingly, this effect could only be shown in male piglets, whereas females remained unaffected.

Similar to TNF-α, IL-10 concentrations reached their maximum 1 h after LPS application and were significantly different between male C and DG pigs. This is surprising, as we expected either the same cytokine concentrations in both deprivation groups, as seen for TNF-α, or that DG pigs would be closer to C pigs, assuming that maternal deprivation with littermates would be less stressful than a total deprivation. A possible explanation for the lower concentrations in the DG group might be increased stress due to regrouping. The random assignment of piglets to either the DA or the DG group was carried out regardless of dominance hierarchies. The regrouping of the five littermates in the deprivation box could have provoked fights within the DG group to establish a new group hierarchy and thus caused more severe stress than being isolated. However, this is speculative because it was not possible to perform behavioural observations during the deprivation procedures. To assess innate immune responses to bacterial challenges, the ratio of pro- and anti-inflammatory cytokines may be even more important than their individual concentrations. A dysbalance of this ratio is thought to cause depression and burn-out symptoms [49,50]. Here, we analysed the ratio of the pro-inflammatory cytokine TNF-α to the anti-inflammatory cytokine IL-10. The male pigs of the DA group had a lower TNF-α/IL-10 ratio compared to the controls but also compared to the DG pigs, which were similar to the controls. A higher ratio implies a stronger response, which could be more appropriate to fight a bacterial infection. Thus, the presence of littermates may reduce the negative effects of social stress, at least in males.

Strikingly, TNF-α and IL-6 concentrations as well as the TNF-α/IL-10 ratios were significantly lower in the females than in the males of the control group. This is in line with a human study in which males exhibited higher concentrations of TNF-α, IL-6 and IL-1β while the IL-10 concentrations did not differ between the sexes [51]. There is evidence that in males, peripheral blood mononuclear cells produce more TNF-α in response to LPS than in females [52,53]. However, while the prior deprivation treatment caused a significantly lower cytokine response in males, this effect was not found in females, which is in line with a study on prenatally stressed pigs [54]. Other studies suggest that a different regulation of the HPA axis in males and females and therefore an altered adaptation strategy to stressful situations might be the reason [55]. A number of studies dealt with the effects of sex on stress and immune responses. For example, Rohleder et al. (2001) found that men exhibited significantly diminished TNF-α and IL-6 secretion in response to LPS when previously exposed to psychosocial stress while it remained unchanged in women [33]. While most of these studies were performed in adults showing fully developed sexual dimorphism, only two studies in 6-week old piglets showed sex differences in response to low-dose LPS treatment with male piglets having higher TNF-α [47,54], which is consistent with our findings. To date, there is a lack of studies considering sex-dependent effects of psychosocial stress on immunity during the neonatal period or childhood, where the influence of sex hormones is not yet very strong. In addition to the activation of the immune system by the increased release of cytokines, the LPS application also induced a significant increase in plasma cortisol concentrations 3 h post-injection. The release of glucocorticoids following the elevation of pro-inflammatory cytokines is part of an inhibitory mechanism to prevent the immune system from overreaction. However, the prior deprivation treatment in the present study did not alter glucocorticoid release in response to LPS, nor did it differ between males and females, which is consistent with the findings in other studies [35,41]. 

The regulatory mechanisms of the HPA axis are quite complex and depend on many different factors and tissues. The hypothalamus plays a major role in the regulation and release of glucocorticoids [56]. In turn, the hypothalamus is influenced by input from limbic areas such as the amygdala, which is involved in the emotional processing of psychosocial stressors [57]. Maternal deprivation is predominantly a psychosocial stressor [58], and its concomitant emotional stress can cause the release of glucocorticoids [27,59]. As mediators of HPA axis-related communication, central cytokines may regulate inflammatory reactions, sickness symptoms such as reduced appetite and fever as well as the activation of the HPA axis [60]. The deprivation procedure in the present study caused significantly lower TNF-α mRNA expression in the hypothalamus of male DG piglets in response to LPS, which corresponds to lower peripheral TNF-α plasma concentrations. Furthermore, we found that prior maternal deprivation caused significantly lower MR mRNA expression in the amygdala in response to LPS, but only in the male DG piglets. The MR responds to basal glucocorticoid levels and is highly important for the maintenance of homeostasis. A change in the MR concentration could change neuronal excitability, which in turn may also affect stress responsiveness, homeostasis and behaviour [61,62]. In addition, GR mRNA expression tended to be lower in the amygdala of DA and DG pigs than in the controls. Similar results were found in previous studies in pigs where exposure to stress caused significantly reduced MR mRNA expression and a reduced MR/GR mRNA ratio in the amygdala [36,63]. However, in that study, the pigs were exposed only once to 4 h of isolation, and no sex difference was found. In the present study, we found sex differences in MR mRNA expression and the MR/GR mRNA ratio, which were significantly higher in males than in females. Interestingly, we found higher IL-10 mRNA expression in the amygdala of females than in males. Assuming that centrally produced IL-10 may counteract inflammatory cytokines [64] this may explain why females in the present study exhibited lower inflammatory cytokine concentrations in the periphery (TNF-α and IL-6) than males in response to LPS. In rats, IL-10 has been shown to have a protective effect on neurons after LPS treatment but also after brain injuries [65,66]. However, the deprivation treatment caused a significant reduction in IL-10 mRNA expression in the amygdala of females in both treatment groups compared to the controls. Thus, psychosocial stress seems to affect the sexes in different manners. This is in line with studies describing different disease susceptibilities depending on sex [67]. For instance, females show a higher resistance to infections [68,69,70] but in turn are more vulnerable to mood disorders and autoimmune diseases [67,71,72]. Nonetheless, in our study we found programming effects of the stress treatment, whereas the underlying mechanisms are not yet clear. Recently, there has been growing interest in differential sensitivity of brain development to early-life adversity in males and females, which may explain sex-specific long-term effects on emotional and cognitive behaviour and the timing of sexual maturation [73]. Therefore, further studies should investigate epigenetic modifications or neuromorphological changes, as these mechanisms are believed to determine the long-term effects of stress [74,75,76]. 

To our knowledge, this is the first study describing the sex-specific effects of psychosocial stress on the innate immunity of pre-pubertal pigs. In stressed males, psychosocial stress affected both peripheral and central pro-inflammatory cytokine responses, whereas in stressed females, it altered the central anti-inflammatory cytokine responses to LPS. In conclusion, our study suggests that psychosocial stress experienced during the neonatal period sensitises the neuroendocrine-immune network and may have sex-specific programming effects on immune responses with consequences for health and welfare. Given the physiological similarities between pigs and humans, our study indicates that pig models could be used to investigate sex differences in the effects of early postnatal stress.

## 4. Materials and Methods 

### 4.1. Animals and Experimental Design

All procedures involving animal handling and treatment were conducted in strict accordance with the German Animal Protection law and were approved by the relevant authorities (Landesamt für Landwirtschaft, Lebensmittelsicherheit und Fischerei, Mecklenburg-Vorpommern, Germany; LALLF M-V/TSD/7221.3-1.1-003/18).

A total of 200 piglets were obtained from 20 litters (German Landrace) born and raised in the experimental pig unit of the Leibniz Institute of Farm Animal Biology (Dummerstorf, Germany). After birth, the litter size was standardised to ten piglets to provide optimal and equal lactation conditions. The pigs were used in ten trials and in each trial two litters were randomly assigned to a deprivation and control litter. During the suckling period, sows and their piglets were housed in a separate loose farrowing pen (6 m^2^) with a plastic floor covered with saw-dust and a water-heated lying area for the piglets with a nearly constant temperature (28 ± 1 °C), an automatic ventilation system and controlled lighting (12/12 h light/dark cycle, lights on at 0600 h). The sows had unrestricted access to food and water. The piglets had unrestricted access to water and were offered feed in addition to milk starting from day 14 of age ((HAKRA-Immuno-G; Una Hakra, Hamburg, Germany). On the first day of life, each piglet received one dose of iron paste (PUCORAL^®^ FerroPlus; Pulte, Grünwald, Germany) and an iron injection at postnatal day 10 (Ursoferran, 2 mL, 100 mg/mL, Serumwerk Bernburg AG, Bernburg, Germany). The piglets were not subjected to tail docking, teeth clipping or castration of the males. The piglets were weaned at 4 weeks by removal of the sow in a mixed group from the deprivation and control litter and placed in weaning pens with an automatic ventilation system under controlled temperature and lighting conditions (12/12 h light/dark cycle, lights on at 0600 h, with a room temperature of 28 ± 1 °C in the first days after weaning and a continuous decrease to 22 ± 1 °C up to an age of 6 weeks). They were offered a commercially pelleted diet from an automatic feeder. Food and water were provided ad libitum.

In the litters assigned to maternal deprivation, half of the piglets was randomly assigned to each of the two social stress procedures with an approximately equal sex ratio: (1) maternal and littermate deprivation, i.e., a total social isolation (5 piglets were separated alone, DA); and (2) maternal and partial littermate deprivation (5 piglets were separated as a group, DG). On days 2–15 of age, the piglets of both treatment groups were deprived for 2 h in the morning (0700-0900 h) in separate test rooms located within the same experimental station. During the social deprivation period, the piglets were placed in special opaque boxes either alone (60 × 40 × 32 cm) or as a familiar group (159 × 68 × 56 cm) with sawdust on the floor and adequate air passage. The socially deprived piglets were kept under the same air and temperature conditions as in the farrowing pen. The piglets of the control litter (C) remained undisturbed in the farrowing pen during this time. The health status of the piglets was checked continuously by visual inspection (general appearance, feeding/drinking behaviour, activity, gait and posture abnormalities) throughout the testing period. None of the piglets showed any clinical signs of disease.

To investigate the effects of different social deprivation treatments on behavioural and physiological responses to an endotoxin challenge, LPS was applied 4 weeks after the deprivation period (day 42) to 20 piglets of each treatment group (60 piglets in total). This day was chosen because piglets habituated to weaning, and the efficiency of their immune system is comparable to that of adults [77]. Two randomly assigned piglets from each treatment group (DA, DG, and C) were intraperitoneally injected with 50 µg/Kg body weight LPS (*Escherichia coli* O111:B4; Sigma-Aldrich, Deisenhofen, Germany).

### 4.2. Behavioural Observations

After the LPS application, piglets were placed back in their home pen and directly observed for 6 h by scan sampling every 5 min to determine the presence of the following sickness symptoms: (1) somnolence (piglets lay separately in a drowsy state with both eyes closed), (2) panting (wheezing noises), (3) circulatory difficulties (rubor of the whole body, cyanosis of nose and ears, balance disorders in gait or posture), (4) shivering (piglets displayed rapid, synchronous muscle contractions, frequently accompanied by piloerection), (5) salivation (saliva discharge from the snout), (6) empty chewing (jaw movement without feed), (7) vomiting (retching and expulsion of the stomach contents), and (8) diarrhoea (semi-liquid or watery faeces). General behaviour observations included activity (moving, exploration, feeding, drinking) and inactivity (lying with or without body contact, sitting, standing). All observations were carried out by a trained person who was blinded to the social deprivation treatment. Rectal temperatures were measured with a commercially available digital thermometer to an accuracy of ±0.1 °C (PRT 2000 Age Precision; Braun GmbH, Kronberg, Germany) before and 1, 3, 6 and 24 h after LPS application.

### 4.3. Blood and Tissue Sampling

Blood samples were taken while piglets were in a supine position by anterior vena cava puncture (the whole procedure lasted approx. 1 min) before LPS application and 1, 3, 6 and 24 h afterwards. The samples were transferred to ice-cooled polypropylene tubes containing EDTA solution, placed on ice and subsequently centrifuged at 2000× *g* for 15 min at 4 °C. Plasma was then stored at −20 °C until analysis. After the last blood sample, piglets were anaesthetised with Ursotamin^®^ (100 mg/mL ketamine hydrochloride, Serumwerk Bernburg AG, Bernburg, Germany) and Stresnil^®^ (40 mg/mL Azaperone, Elanco, Homburg, Germany) and killed by an intravenous injection of T61^®^ (embutramide/mebezonium iodide/tetracaine hydrochloride, Intervet, Unterschleißheim, Germany). The brains were quickly removed and the hypothalamus and amygdala were dissected from both hemispheres and stored at 80 °C until mRNA analysis. All experimental procedures were performed between 0800 and 1100 h. Because of technical problems, only 16 plasma samples and 14 tissue samples of each brain region per treatment group could be analysed.

### 4.4. Cytokine and Hormone Assays

Plasma cortisol concentrations were measured in duplicate using a commercially available ELISA kit (DRG instruments, Marburg, Germany) according to the manufacturer’s instructions. The assay was validated for use with porcine plasma as previously described [78]. The sensitivity of the assay was 3.4 ng/mL, and the intra- and inter-assay coefficients of variation (CV) were 6.2% and 9.4%, respectively. 

The concentrations of TNF-α, IL-10 and IL-6 were analysed in duplicate in plasma samples by commercially available pig ELISA kits (R&D Systems, Minneapolis, USA) according to the manufacturer’s instructions. The sensitivities of the TNF-α and IL-10 assays were 3 pg/mL. The intra- and inter-assay CVs of the TNF-α assay were 6.2% and 8.2%, respectively and those of IL-10 were 6.3% and 9.4%, respectively. The sensitivity of the IL-6 assay was 3.8 pg/mL, and the intra- and inter-assay CVs were 4.0% and 5.9%, respectively.

### 4.5. RNA Extraction and Quantification of Transcripts

RNA extraction of brain samples was performed using the RNeasy Lipid Tissue Kit (Quiagen, Hilden, Germany) according to the manufacturer’s protocol. The RNA concentration was determined at 260 nm by the use of a NanoPhotometer^TM^ (Implen, München, Germany) and the purity and integrity were determined by calculating the 260/280 nm ratio. mRNA expression was monitored by reverse transcription (RT) of 750 ng of RNA using the iScript cDNA synthesis kit (Bio-Rad, München, Germany) according to the manufacturer’s guidelines. The resulting cDNA was amplified by real-time PCR (iCycler, Bio-Rad, München, Germany) for the following genes: *NR3C2* (mineralocorticoid receptor; MR), *NR3C1* (glucocorticoid receptor; GR), *CRHR1* (corticotropin releasing hormone receptor 1), *CRHR2* (corticotropin releasing hormone receptor 2), *TNFA* (tumour necrosis factor-alpha), *IL6* (interleukin-6) and *IL10* (interleukin-10). One microlitre of the RT reaction solution was added to 6 µL of iQ SYBR Green Supermix (Bio-Rad, München, Germany) and 4 µL of primer mix with gene-specific oligonucleotides (TIB Molbiol, Berlin, Germany). All reactions were performed in triplicate. Primers were designed corresponding to the gene sequences of the NCBI database. Whenever possible, primers were designed to span the exon-exon junctions and to anneal between 57 and 61 °C. The oligonucleotide sequences of the primers are summarised in Table 5.

PCR was performed using a hot start (3 min, 94 °C; 30 s, 60 °C; 45 s, 70 °C), 39 cycles (10 s 94 °C; 30 s 60 °C; 45 s 70 °C with 5 s of time extension per cycle) and a final cycle (10 s 94 °C; 30 s 60 °C; 7 min 70 °C, 1 min 94 °C), corresponding to denaturation, annealing and elongation respectively. The specificity of the products was assessed using melting point analysis (60 °C to 90 °C, 1 °C per 10 s), and agarose gel electrophoresis (3.5%). The oligonucleotide structure was verified by sequencing in a subset of the experiments. The relative quantification was calculated using the quantification module of the CFX Manager Software^TM^ version 2.1 (Bio-Rad, München, Germany). Data for mRNA expression of the investigated genes are presented as relative expression ratios normalised to *ACTB* (beta-actin) and *TBP* (TATA-box binding protein).

### 4.6. Statistical Analysis

Statistical analysis was performed using SAS software for Windows, version 9.4 (Copyright, SAS Institute Inc., Cary, NC, USA). Descriptive statistics and tests for normality were calculated with the UNIVARIATE procedure of the Base SAS software.

Plasma data could be considered as approximately normal and were analysed by repeated-measures analysis of variance (ANOVA) with the GLIMMIX procedure of SAS/STAT software using a normal model with the fixed effects social treatment (levels: DA, DG, C), sex (levels: female, male), time (levels: 0 h, 1 h, 3 h, 6 h, 24 h) as repeated variables, trial (levels: 1–10) and the treatment × sex, treatment × time and treatment × time × sex interactions. Sow was included as a random effect. Count data of sickness behaviour were analysed by the GLIMMIX procedure using a Poisson model with the fixed effects social treatment (levels: DA, DG, C), sex (levels: female, male), time (levels: 1 h, 2 h, 3 h, 4 h, 5 h, 6 h) as repeated variables, trial (levels: 1–10) and the treatment × sex, treatment × time and treatment × time × sex interactions. Sow was included as a random effect. Least squares means (LS means) and standard errors (SE) were calculated for each fixed effect of the normal and Poisson models, and multiple pairwise comparisons of these LS means were performed with the Tukey-Kramer procedure.

Gene expression data were analysed by ANOVA using the GLIMMIX procedure and a model with treatment (levels: DA, DG, C), sex (levels: female, male), trial (levels: 1–10) and the treatment × sex interaction as fixed effects and sow as a random effect. Least squares means (LS means) and standard errors (SE) were calculated for each fixed effect of the model and multiple pairwise comparisons of these LS means were performed with the Tukey-Kramer procedure. Differences were considered significant if *p* ≤ 0.05.

## Figures and Tables

**Table 1:** Occurrences of parameters indicative of sickness behaviour in piglets of different treatment groups after LPS application.

	Treatment Group			*p*-Value (F-Test)				
Parameter	DA	DG	C	Treatment	Time	Sex	Treatment × Time × Sex	Treatment × Time
*Somnolence (counts)*				<0.05	<0.001	0.563	0.871	0.804
1 h	5.45 ± 0.40	5.20 ± 0.40	4.70 ± 0.40					
2 h	8.55 ± 0.40	8.85 ± 0.40 ^b^	7.25 ± 0.40 ^a^					
3 h	10.85 ± 0.40	10.60 ± 0.40	10.45 ± 0.40					
4 h	9.75 ± 0.40	9.95 ± 0.40	9.45 ± 0.40					
5 h	11.25 ± 0.40	11.15 ± 0.40	10.75 ± 0.40					
6 h	11.30 ± 0.40	10.80 ± 0.40	10.85 ± 0.40					
*Panting (counts)*				<0.05	<0.001	0.242	0.635	0.060
1 h	1.05 ± 0.66	1.05 ± 0.66	0.25 ± 0.66					
2 h	5.95 ± 0.66 ^b^	5.15 ± 0.66 ^b^	2.70 ± 0.66 ^a^					
3 h	7.70 ± 0.66 ^b^	7.55 ± 0.66 ^b^	4.95 ± 0.66 ^a^					
4 h	3.00 ± 0.66	4.60 ± 0.66	2.85 ± 0.66					
5 h	1.05 ± 0.66	2.05 ± 0.66	1.15 ± 0.66					
6 h	0.25 ± 0.66	1.75 ± 0.66	0.05 ± 0.66					
*Circulatory problems (counts)*				0.393	<0.001	0.729	0.988	0.922
1 h	2.30 ± 0.61	2.05 ± 0.61	1.25 ± 0.61					
2 h	7.95 ± 0.61	8.55 ± 0.61	7.10 ± 0.61					
3 h	6.85 ± 0.61	7.50 ± 0.61	7.30 ± 0.61					
4 h	2.80 ± 0.61	4.00 ± 0.61	3.60 ± 0.61					
5 h	0.00 ± 0.61	0.65 ± 0.61	0.35 ± 0.61					
6 h	0.00 ± 0.61	0.35 ± 0.61	0.00 ± 0.61					
*Shivering (counts)*				0.174	<0.001	0.846	0.933	0.099
1 h	2.65 ± 0.51	1.75 ± 0.51	1.10 ± 0.51					
2 h	5.10 ± 0.51	4.10 ± 0.51	3.65 ± 0.51					
3 h	4.20 ± 0.51	2.75 ± 0.51	3.70 ± 0.51					
4 h	1.15 ± 0.51	1.30 ± 0.51	2.80 ± 0.51					
5 h	0.75 ± 0.51	0.30 ± 0.51	1.05 ± 0.51					
6 h	0.05 ± 0.51	0.00 ± 0.51	0.25 ± 0.51					
*Salivating (counts)*				0.961	<0.001	0.250	0.959	0.991
1 h	0.40 ± 0.13	0.35 ± 0.13	0.25 ± 0.13					
2 h	0.65 ± 0.13	0.55 ± 0.13	0.65 ± 0.13					
3 h	0.10 ± 0.13	0.25 ± 0.13	0.20 ± 0.13					
4 h	0.00 ± 0.13	0.10 ± 0.13	0.00 ± 0.13					
5 h	0.00 ± 0.13	0.00 ± 0.13	0.00 ± 0.13					
6 h	0.00 ± 0.13	0.00 ± 0.13	0.00 ± 0.13					
*Empty chewing (counts)*				0.616	<0.001	0.936	0.293	0.924
1 h	0.30 ± 0.13	0.60 ± 0.13	0.40 ± 0.13					
2 h	0.50 ± 0.13	0.60 ± 0.13	0.50 ± 0.13					
3 h	0.20 ± 0.13	0.35 ± 0.13	0.45 ± 0.13					
4 h	0.10 ± 0.13	0.00 ± 0.13	0.00 ± 0.13					
5 h	0.00 ± 0.13	0.05 ± 0.13	0.00 ± 0.13					
6 h	0.00 ± 0.13	0.00 ± 0.13	0.00 ± 0.13					
*Vomiting (counts)*				0.453	<0.001	0.920	<0.05	0.947
1 h	0.50 ± 0.22	0.70 ± 0.22	0.55 ± 0.22					
2 h	1.20 ± 0.22	1.45 ± 0.22	1.25 ± 0.22					
3 h	0.60 ± 0.22	1.05 ± 0.22	1.00 ± 0.22					
4 h	0.35 ± 0.22	0.20 ± 0.22	0.05 ± 0.22					
5 h	0.05 ± 0.22	0.25 ± 0.22	0.00 ± 0.22					
6 h	0.00 ± 0.22	0.00 ± 0.22	0.00 ± 0.22					
*Diarrhoea (counts)*				0.591	<0.05	0.408	0.723	0.848
1 h	0.20 ± 0.09	0.00 ± 0.09	0.00 ± 0.09					
2 h	0.25 ± 0.09	0.15 ± 0.09	0.10 ± 0.09					
3 h	0.20 ± 0.09	0.35 ± 0.09	0.20 ± 0.09					
4 h	0.10 ± 0.09	0.10 ± 0.09	0.00 ± 0.09					
5 h	0.00 ± 0.09	0.00 ± 0.09	0.05 ± 0.09					
6 h	0.00 ± 0.09	0.00 ± 0.09	0.00 ± 0.09					
*Activity (counts)*				0.128	<0.001	0.230	0.690	0.765
1 h	2.15 ± 0.33 ^a^	3.15 ± 0.33	3.50 ± 0.33 ^b^					
2 h	0.05 ± 0.33	0.20 ± 0.33	0.40 ± 0.33					
3 h	0.10 ± 0.33	0.05 ± 0.33	0.20 ± 0.33					
4 h	0.10 ± 0.33	0.20 ± 0.33	0.25 ± 0.33					
5 h	0.65 ± 0.33	0.60 ± 0.33	1.05 ± 0.33					
6 h	0.60 ± 0.33	0.45 ± 0.33	1.00 ± 0.33					
*Inactivity (counts)*				0.088	<0.001	0.295	0.930	0.487
1 h	11.20 ± 0.23	10.45 ± 0.23	10.45 ± 0.23					
2 h	11.95 ± 0.23	11.95 ± 0.23	11.80 ± 0.23					
3 h	11.90 ± 0.23	11.95 ± 0.23	11.80 ± 0.23					
4 h	11.95 ± 0.23	11.95 ± 0.23	11.90 ± 0.23					
5 h	11.75 ± 0.23	11.75 ± 0.23	11.70 ± 0.23					
6 h	11.95 ± 0.23	11.15 ± 0.23	11.70 ± 0.23					
*Rectal temp. (°C)*				0.517	<0.001	0.073	0.760	0.359
0 h	39.06 ± 0.23	39.12 ± 0.23	39.11 ± 0.23					
1 h	39.46 ± 0.23	39.40 ± 0.23	39.53 ± 0.23					
3 h	38.69 ± 0.23	38.77 ± 0.23	38.62 ± 0.23					
6 h	39.26 ± 0.23	39.25 ± 0.23	39.85 ± 0.23					
24 h	40.14 ± 0.23	39.55 ± 0.23	40.18 ± 0.23					

DA (deprivation alone), DG (deprivation with a group of littermates) and C (control, no deprivation). Data are expressed as LS means ± SE. within a row, significant differences are indicated by different superscript letters (at least *p* < 0.05; Tukey-Kramer test; *n* = 20 pigs per treatment group).

**Table 2:** Endocrine and immune parameters in piglets of different treatment groups.

	Treatment Group			*p*-Value (F-Test)					
Parameter	DA	DG	C	Treatment	Time	Sex	Treatment × Time × Sex	Treatment × Time	Treatment × Sex
Cortisol (ng/mL)				0.614	<0.001	0.311	0.882	0.543	0.870
0 h	32.50 ± 10.41	31.79 ± 10.81	35.27 ± 10.81						
1 h	92.80 ± 10.42	75.96 ± 10.81	88.16 ± 10.81						
3 h	218.61 ± 10.42	191.87 ± 10.81	186.62 ± 10.81						
6 h	98.21 ± 10.42	107.90 ± 10.81	102.11 ± 10.81						
24 h	26.24 ± 10.42	20.70 ± 10.81	22.10 ± 10.81						
									
TNF-α (ng/mL)				0.127	<0.001	0.354	<0.01	< 0.05	0.094
0 h	0.11 ± 1.85	0.05 ± 1.92	0.05 ± 1.92						
1 h	15.09 ± 1.85 ^b^	21.20 ± 1.92	27.53 ± 1.92 ^a^						
3 h	5.30 ± 1.85	4.96 ± 1.92	5.48 ± 1.92						
6 h	1.25 ± 1.85	1.18 ± 1.92	1.36 ± 1.92						
24 h	0.12 ± 1.85	0.06 ± 1.92	0.07 ± 1.92						
									
IL-6 (ng/mL)				0.837	<0.001	0.575	0.920	0.998	0.600
0 h	0.01 ± 0.34	0.02 ± 0.36	0.02 ± 0.36						
1 h	0.09 ± 0.34	0.14 ± 0.36	0.22 ± 0.36						
3 h	2.21 ± 0.34	2.35 ± 0.36	2.75 ± 0.36						
6 h	0.33 ± 0.34	0.43 ± 0.36	0.43 ± 0.36						
24 h	0.04 ± 0.34	0.04 ± 0.36	0.05 ± 0.36						
									
IL-10 (pg/mL)				0.681	<0.001	0.354	0.180	0.919	0.771
0 h	22.99 ± 8.50	20.80 ± 8.74	26.93 ± 8.76						
1 h	93.04 ± 8.50	97.12 ± 8.74	111.87 ± 8.76						
3 h	23.30 ± 8.50	18.67 ± 8.74	20.09 ± 8.76						
6 h	54.37 ± 8.50	47.10 ± 8.74	58.81 ± 8.76						
24 h	50.85 ± 8.50	55.16 ± 8.74	56.76 ± 8.76						
									
TNF-α/IL-10 ratio				0.231	<0.001	0.067	0.278	0.191	0.721
0 h	7.87 ± 16.42	13.69 ± 17.04	10.03 ± 17.04						
1 h	155.96 ± 16.42	217.86 ± 17.04	237.24 ± 17.04						
3 h	10.60 ± 16.42	8.34 ± 17.04	9.33 ± 17.04						
6 h	97.47 ± 16.42	123.87 ± 17.04	106.60 ± 17.04						
24 h	26.90 ± 16.42	26.18 ± 17.04	27.96 ± 17.04						

DA (deprivation alone), DG (deprivation with a group of littermates) and C (control, no deprivation). Data are expressed as LS means ± SE. Within a row, significant differences are indicated by different superscript letters (at least *p* < 0.05; Tukey-Kramer test; *n* = 16 pigs per treatment group).

**Table 3:** Relative mRNA expression of HPA-related parameters and cytokines in the hypothalamus of piglets of the different treatment groups.

	Treatment Group			*p*-Value (F-Test)		
Parameter	DA	DG	C	Treatment	Sex	Treatment × Sex
MR	1.04 ± 0.20	0.84 ± 0.20	1.12 ± 0.19	0.595	0.058	0.423
GR	1.01 ± 0.23	0.93 ± 0.23	1.16 ± 0.22	0.772	0.517	0.926
CRHR1	1.13 ± 0.18	1.03 ± 0.18	1.24 ± 0.17	0.711	0.828	0.188
CRHR2	0.95 ± 0.19	1.05 ± 0.19	1.23 ± 0.18	0.567	0.874	0.841
TNF-α	1.02 ± 0.19 ^b^	0.83 ± 0.19 ^b^	1.77 ± 0.18 ^a^	<0.01	0.072	0.623
IL-6	1.36 ± 0.20	0.95 ± 0.20	1.35 ± 0.19	0.260	0.841	0.430
IL-10	0.70 ± 0.37	0.23 ± 0.37	0.77 ± 0.37	0.436	0.450	0.252
MR/GR ratio	1.75 ± 1.21	1.06 ± 1.21	2.80 ± 1.17	0.589	0.104	0.456

DA (deprivation alone), DG (deprivation with a group of littermates) and C (control, no deprivation); MR (mineralocorticoid receptor; *NR3C2*), GR (glucocorticoid receptor; *NR3C1*), CRHR1/CRHR2 (corticotropin releasing hormone receptor 1/2), TNF-α (tumour necrosis factor-alpha; *TNFA*), IL-6 (interleukin-6; *IL6*), IL-10 (interleukin-10; *IL10*). Data are expressed as LS means ± SE. Within a row, significant differences are indicated by different superscript letters (at least *p* < 0.05; Tukey-Kramer test; *n* = 14 pigs per treatment group).

**Table 4:** Relative mRNA expression of HPA-related parameters and cytokines in the amygdala of piglets of the different treatment groups.

	Treatment Group			*p*-Value (F-Test)		
Parameter	DA	DG	C	Treatment	Sex	Treatment × Sex
MR	1.09 ± 0.11	0.70 ± 0.12 ^b^	1.42 ± 0.11 ^a^	<0.001	<0.05	0.211
GR	1.10 ± 0.17	1.09 ± 0.18	1.70 ± 0.17	<0.05	0.567	0.965
CRHR1	1.32 ± 0.13	1.14 ± 0.14	1.44 ± 0.13	0.332	0.104	0.600
CRHR2	1.05 ± 0.11	0.82 ± 0.11	0.97 ± 0.11	0.272	0.291	0.387
TNF-α	1.11 ± 0.17	0.94 ± 0.18	1.42 ± 0.17	0.162	0.856	0.670
IL-6	1.11 ± 0.28	0.72 ± 0.29	1.39 ± 0.28	0.167	0.424	0.577
IL-10	1.35 ± 0.22 ^b^	0.87 ± 0.22 ^b^	2.01 ± 0.22 ^a^	<0.01	<0.05	<0.05
MR/GR ratio	1.03 ± 0.15	0.88 ± 0.16	0.95 ± 0.15	0.782	<0.05	0.507

DA (deprivation alone), DG (deprivation with a group of littermates) and C (control, no deprivation); MR (mineralocorticoid receptor; *NR3C2*), GR (glucocorticoid receptor; *NR3C1*), CRHR1/CRHR2 (corticotropin releasing hormone receptor 1/2), TNF-α (tumour necrosis factor-alpha; *TNFA*), IL-6 (interleukin 6; *IL6*), IL-10 (interleukin-10; *IL10*). Data are expressed as arbitrary units after normalisation to ACTB and TBP mRNA expression as endogenous reference genes and represent the LS means ± SE. Within a row, significant differences are indicated by different superscript letters (at least *p* < 0.05; Tukey-Kramer test; *n* = 14 pigs per treatment group).

**Table 5:** Genes, primer sequences and amplicon sizes.

Gene	GeneBank Accession Numbers	Sense, Antisense Primer (5′–3′)	Amplicon (bp)
MR	ENSSSCG00000037766	AGTGTTCTTCAAAAGAGCAGTGG, CCTCGTGGATCCCTTTCAAC	188
GR	NM_001008481.1	GTTCCAGAGAACCCCAAGAGTTCA, TCAAAGGTGCTTTGGTCTGTGGTA	173
CRHR1	NM_001144110.1	CTCATCTCAGCCTTCATCCTG, CGAACATCCAGAAGAAGTTGG	151
CRHR2	NM_001144118.1	CAGGGTTTCTTCGTGTCTGTC, GTCTGCTTGATGCTGTGGAAG	173
TNF-α	NM_214022.1	TCCTCACTCACACCATCAGC, TAGTCGGGCAGGTTGATCTC	199
IL-6	NM_214399.1	TGCTTCTGGTGATGGCTACTG, TTCTGCCAGTACCTCCTTGC	209
IL-10	NM_214041	AGCCAGCATTAAGTCTGAGAAC, CCTCTCTTGGAGCTTGCTAA	394
ACTB ^*^	ENSSSCT00000042531	TCTGGCACCACACCTTCT, TGATCTGGGTCATCTTCTCAC	114
TBP ^*^	NM_003194.5	AACAGTTCAGTAGTTATGAGCCAGA, AGATGTTCTCAAACGCTTCG	153

MR (mineralocorticoid receptor; *NR3C2*), GR (glucocorticoid receptor; *NR3C1*), CRHR1/CRHR2 (corticotropin releasing hormone receptor 1/2), TNF-α (tumour necrosis factor-alpha; *TNFA*), IL-6 (interleukin-6; *IL6*), IL-10 (interleukin-10; *IL10*), *ACTB* (actin beta), *TBP* (TATA-box binding protein); * reference gene.

## Abbreviations

C	Control pigs
CD14	Cluster of differentiation 14
cDNA	Complementary DNA
CNS	Central nervous system
CRHR1	Corticotropin-releasing hormone receptor 1
CRHR2	Corticotropin-releasing hormone receptor 2
CV	Coefficients of variance
DA	Deprivation alone
DG	Deprivation in a group of littermates
DNA	Deoxyribonucleic acid
EDTA	Ethylenediaminetetraacetic acid
GR	Glucocorticoid receptor (gene-name NR3C1)
HPA	Hypothalamic-pituitary-adrenal axis
IL-1β	Interleukin-1 beta
IL-6	Interleukin-6
IL-10	Interleukin-10
LPS	Lipopolysaccharide
LS mean	Least squares mean
MR	Mineralocorticoid receptor
mRNA	Messenger RNA
NF-кB	Nuclear factor kappa B
NR3C1	Nuclear receptor subfamily 3 group C member 1 (alias GR)
NR3C2	Nuclear receptor subfamily 3 group C member 2 (alias MR)
PCR	Polymerase chain reaction
qPCR	Quantitative polymerase chain reaction
RNA	Ribonucleic acid
SE	Standard error
TLR4	Toll-like receptor 4
TNF-α	Tumour necrosis factor-alpha
